# Structure determination of a low-crystallinity covalent organic framework by three-dimensional electron diffraction

**DOI:** 10.1038/s42004-023-00915-4

**Published:** 2023-06-07

**Authors:** Guojun Zhou, Taimin Yang, Zhehao Huang

**Affiliations:** Department of Materials and Environmental Chemistry, Stockholm University, Stockholm, SE-106 91 Sweden

**Keywords:** Analytical chemistry, Characterization and analytical techniques, Polymer chemistry, Porous materials

## Abstract

Covalent organic frameworks (COFs) have been attracting intense research due to their permanent porosity, designable architecture, and high stability. However, COFs are challenging to crystallize and their synthesis often results in tiny crystal sizes and low crystallinities, which hinders an unambiguous structure determination. Herein, we demonstrate that the structure of low-crystallinity COF Py-1P nanocrystals can be solved by coupling three-dimensional electron diffraction (3DED) with simulated annealing (SA). The resulting model is comparable to that obtained from high-crystallinity samples by dual-space method. Moreover, for low-resolution 3DED data, the model obtained by SA shows a better framework than those provided by classic direct method, dual-space method, and charge flipping. We further simulate data with different resolutions to understand the reliability of SA under different crystal quality conditions. The successful determination of Py-1P structure by SA compared to other methods provides new knowledge for using 3DED to analyze low-crystallinity and nanosized materials.

## Abstract (web summary)

Covalent organic frameworks have demonstrated promise for a wide variety of applications, but their synthesis often results in materials with low crystallinity that are challenging to characterize. Here, coupling three dimensional electron diffraction with simulated annealing is shown to enable the structural determination of a low crystallinity pyrene-containing covalent organic framework.

## Introduction

Covalent organic frameworks (COFs) are crystalline porous polymers that are constructed by connecting organic monomers via strong covalent bonds^1, 2^. Their precise integration of organic units at an atomic level to create predesigned framework structures endows them as a promising class of materials in various applications such as gas sorption^3, 4^, separations^5–7^, catalysis^8–11^, sensing^12^, optics^13, 14^, electronics^15–17^, etc. A grand challenge in the development of COFs is to characterize their structures at the atomic level. Single-crystal X-ray diffraction is the common method used to study crystal structures. While different strategies have been developed to obtain COF single-crystals^18, 19^, however, limited by the reaction kinetics and thermodynamics of polymerization, the synthesis of COFs often yields low-crystallinity products with small particle sizes. This hinders the determination of COF structures and prevents a deep understanding of their structure–property relationships.

Three-dimensional electron diffraction (3DED) has been developed as a complementary method to single-crystal X-ray diffraction for determining structures of tiny crystals^20–26^. It has successfully been applied for ab initio determination of COF structures from nanocrystals^27–30^. However, as a single-crystal analysis method, 3DED requires good crystal quality to give sufficient data resolution (<1.2 Å) for ab initio structure determination^31–36^. The classic direct method^37^, dual-space method^38^, charge flipping^39^, and simulated annealing^40, 41^ are common methods that have been used for solving crystal structures. Among these methods, the dual-space method and simulated annealing have been successfully applied for determining COF structures^27, 42^. However, as COFs synthesis often results in low-crystallinity products, it poses a significant challenge due to their low data resolution. Modeling COF structures based on powder X-ray diffraction (PXRD) patterns is a common approach to obtain reasonable COF structures from low-crystallinity samples^43^. However, as COFs usually have large unit cells, which result in severe peak overlapping on PXRD patterns, it is difficult to distinguish different possible models, especially from low-resolution data. Moreover, peak broadening resulted from low crystallinity could further intensify peak overlapping. In contrast, peak overlapping is a petty concern for electron diffraction, because the distance between the crystal and camera can be virtually adjusted by camera length, and thus separate the peaks. For low-crystallinity compounds, although Bragg reflections can be affected by the structural disorder, mosaicity, lattice strain, etc., as long as electron diffraction data include well-defined Bragg reflections, important structural information can be obtained.

While a genetic algorithm has been used for direct-space determination of COF structures with low data resolution^44^, here, we demonstrate another example of coupling a real-space approach simulated annealing^45, 46^ with continuous 3DED to solve the structures of low-crystallinity COFs. Importantly, by comparing the results from a classic direct method, dual-space method, charge flipping, and simulated annealing, we provide an overview of how they perform in dealing with the different data quality in COFs crystallography. Despite the low crystallinity, we demonstrate that the COF structure obtained from SA is comparable to the refined structure obtained from ab initio structure determination by dual-space method, in terms of bond distances and angles. In addition, using simulated 3DED data, we study the resolution limits, from which the COF structure can be reliably obtained by SA.

## Results

**Fig. 1:**
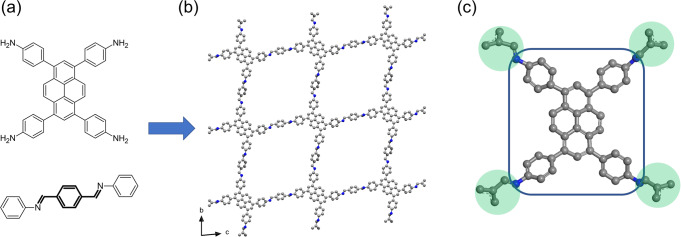
Structure of Py-1P and the fragment used for SA. **a** The organic monomers for building Py-1P. **b** The structural model of Py-1P. **c** The fragment used for SA. Flexibility for rotation is allowed along the C-N bond connecting the benzyl groups and the imine groups. Gray spheres: C; blue spheres: N.

The COF Py-1P single nanocrystals were first reported in our previous study, as a result of understanding COF crystal growth^29^. Py-1P was synthesized by using 4, 4′, 4′′, 4′′′-(1,9-dihydropyrene-1,3,6,8-tetrayl)-tetraaniline (DTA) and aniline-protected aldehyde (1,4-phenylene)bis(*N*-phenylmethanimine) (PPA) as the monomers (Fig. 1a). The crystal structure was first solved by ab initio phasing using the dual-space method^38^, and refined against the 3DED data having a high resolution of 0.9 Å^29^. As the refined structural model provides accurate structural information, we use it as the reference for comparison in our study.

**Fig. 2:**
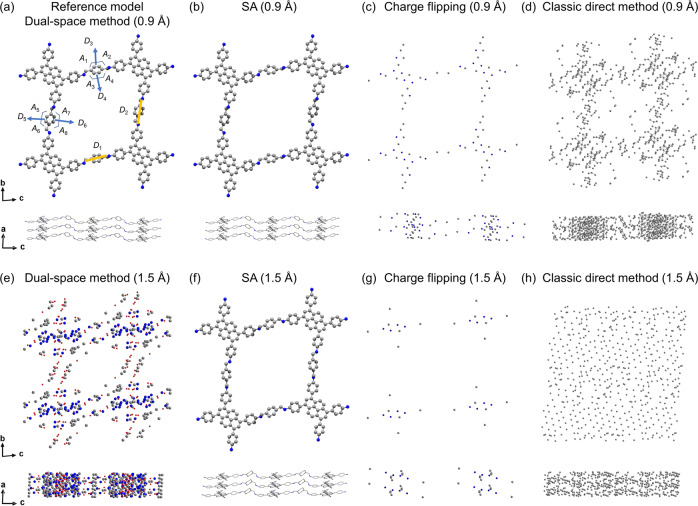
Comparison of the structural models obtained by different methods using 3DED data. **a** The refined reference structural model obtained by the dual-space method. The structural models were obtained using **b** SA, **c** charge flipping, and **d** classic direct method with 0.9 Å resolution. The structural models were obtained using **e** dual-space method, **f** SA, **g** charge flipping, and **h** classic direct method with 1.5 Å resolution. *D*_1_ and *D*_2_ indicate the distances between the N atoms; the *D*_3_, *D*_4_, *D*_5_, and *D*_6_ are the C … C bond distances between two fragments; the *A*_1_, *A*_2_, *A*_3_, *A*_4_, *A*_5_, *A*_6_, *A*_7_, and *A*_8_ are the bond angles involving C atoms from two fragments in the phenyl rings. Gray spheres: C; blue spheres: N; red spheres: O.

To understand the accuracy of using 3DED data for solving COF structures by SA, we first use the same high-resolution dataset (0.9 Å) as it was used for ab initio phasing. The starting model for SA was based on the molecular structures of the monomers, which are optimized by density functional theory (DFT) calculation (Supplementary Fig. 1). As the polymerization reaction between DTA and PPA would result in imine groups^29^, we chose to include the imine groups when building the fragment for SA (Fig. 1c). Specifically, DTA was defined as a rigid body (blue box in Fig. 1c), whose position, including translation and rotation, is refined by SA. Moreover, flexibility for rotating is allowed along the C-N bonds connecting the benzyl groups and the imine groups (the C_4_ groups in green circles in Fig. 1c). After ten runs of SA calculation, with each run including 20 itineraries, the lowest cost function (CF) value based on *R* structure factor^47^ is 0.596. Notably, in selecting the best model from different structural solutions, not only the CF values but also the geometry of the framework should be taken into account. This is particularly important for 3DED data, because electron beams can damage the crystals, and it is difficult to reach 100% data completeness for low-symmetry, i.e., triclinic and monoclinic crystals. We, therefore, compare the bond distances and angles from the best structural model obtained by SA to those in the reference structure. As a rigid body is used in the fragments for SA, we identified the following key distances and angles for comparison (Fig. 2a): (i) The *D*_1_ and *D*_2_ distances between the N atoms in each imine group, (ii) the *D*_3_, *D*_4_, *D*_5_, and *D*_6_ distances showing the C … C bonds in two phenyl rings formed between two fragments; and (iii) the *A*_1_–*A*_8_ angles in the two phenyl rings. For the comparison of *D*_1_ and *D*_2_ distances, we compare the values from the structure solution to those from the refined reference structural model, which was obtained from the best data quality. For the comparison of *D*_3_–*D*_6_ and *A*_1_– *A*_8_, we compare the values from the structure solution to those from standard values of phenyl rings, which is 1.39 Å for C … C bond distance and 120^o^ for C … C … C bond angle. Comparing the reference model and the model obtained by SA, the differences in *D*_1_ and *D*_2_ are 0.12 and 0.01 Å, respectively, which indicates a slight tilting of the fragments. The geometry of the phenyl rings formed between two fragments shows how reasonable the rotation of the C_4_ groups is. The SA model shows a higher degree of distortion in the phenyl rings. Specifically, comparing the bond distances and angles in the phenyl rings from the reference model and the model obtained by SA, the average difference in distance (vs 1.39 Å) increased from 0.01 to 0.12 Å and the average difference in angles (vs 120^o^) increased from 2^o^ to 8^o^ (Table 1). Overall, the structural models show high similarity from the same high-resolution 3DED data (Fig. 2a, b). Thus, it shows SA as a reliable method to solve COF structures, and the distortion in the phenyl rings can be tackled during the refinement.

**Table 1:** Comparison of key bond distances and angles in different structural models.

	Refined reference model (0.9 Å)	SA (0.9 Å)	Difference^a^ (0.9 Å)	SA (1.5 Å)	Difference^a^ (1.5 Å)
*D*_1_ (Å)	7.00(5)	7.12	0.12	6.85	0.15
*D*_2_ (Å)	7.03(5)	7.02	0.01	6.91	0.12
*D*_3_ (Å)	1.39(6)	1.50	0.11	1.79	0.4
*D*_4_ (Å)	1.39(6)	1.56	0.17	1.86	0.47
*D*_5_ (Å)	1.36(6)	1.31	0.08	1.35	0.04
*D*_6_ (Å)	1.40(6)	1.51	0.12	1.32	0.07
*A*_1_ (°)	119(3)	106	14	140	20
*A*_2_ (°)	124(4)	137	17	86	34
*A*_3_ (°)	122(4)	132	12	86	34
*A*_4_ (°)	121(4)	106	14	136	16
*A*_5_ (°)	122(4)	122	2	136	16
*A*_6_ (°)	122(4)	125	5	103	17
*A*_7_ (°)	120(4)	119	1	104	16
*A*_8_ (°)	121(3)	118	2	135	15

^a^Differences between *D*_1_ and *D*_2_ are calculated against the refined reference model. Differences between *D*_3_–*D*_6_ and *A*_1_–*A*_8_ are calculated against optimal values in phenyl rings. All values are shown in absolute values.

Besides SA, other common methods for structure determination of nanocrystals include the classic direct method, such as implemented in SHELXS^48^, the dual-space method, such as implemented in SHELXT^38^ and charge flipping^39^. For the same high-resolution data, we compare the structural models obtained by charge flipping, and the classic direct method to that obtained by SA and the refined reference model obtained by the dual-space method. The structural models obtained by charge flipping and the classic direct method show a similar atomic distribution as the reference model and the SA model, from which the pore of the COF can be identified (Fig. 2c, d). However, charge flipping resulted in a model with lower atomic density, while the classic direct method resulted in a model with higher atomic density than the reference model. As a consequence, both models lack detailed crystallographic information, such as bonding information.

**Fig. 3:**
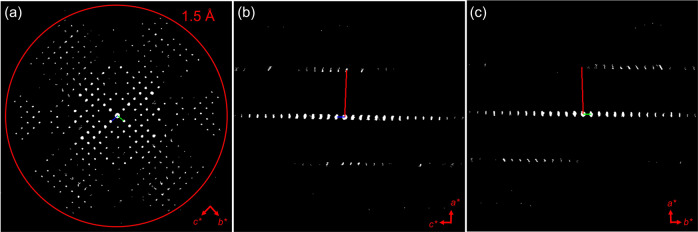
The reconstructed 3D reciprocal space of low-crystalline Py-1P. The 3D reciprocal space is viewed along **a** the [100], **b** the [010], and **c** the [001] directions.

As limited by crystallization processes, many COFs are obtained with crystallinities showing lower data resolution than 0.9 Å. Low-resolution 3DED data can provide crucial information about unit cell parameters and space groups. Combining such information with the intensities extracted from Bragg reflections, the optimum positions of the organic building units can be identified by using SA. During our 3DED data collection, besides the high-quality crystals of Py-1P, we also found less crystallinity samples which have low resolution of c.a. 1.5 Å. We were still able to determine the unit cell parameters and space group from the low-resolution data (Fig. 3), as *a* = 3.9160(2) Å, *b* = 23.760(48) Å, *c* = 23.580(15) Å, *α* = 84.01(22)°, *β* = 88.69(1)°, *γ* = 89.95(2)°, and space group of *P*1, which are similar to those obtained from high-quality crystals (*a* = 3.9280(8) Å, *b* = 23.393(5) Å, *c* = 23.544(5) Å, *α* = 84.51(3)°, *β* = 87.07(3)°, *γ* = 87.05(3)°, and *P*1 as the space group). We then compare different methods, including the dual-space method, SA, charge flipping, and classic direct method to solve the COF structure from the low-resolution data. The same unit cell parameters from the reference data were used. We found that SA can successfully solve the structure from the low-crystallinity COF, though the CF value increased to 0.639. Compared to the reference model, the *D*_1_ and *D*_2_ differ by 0.15 and 0.12 Å, respectively. The distortion in the phenyl rings become more severe, with an average difference of 0.24 Å in bond distance and 21° in bond angles (Table 1). Nevertheless, the interpretation of the framework structure can be successfully concluded (Fig. 2f). In contrast, the other methods failed to obtain a meaningful structural model. Among them, pores can be recognized in the structural model solved by the dual-space method (Fig. 2e). For the models obtained by charge flipping and the classic direct method, the resolved number of atoms is either too low or too high, which prevents the interpretation of the framework (Fig. 2g, h).

**Fig. 4:**
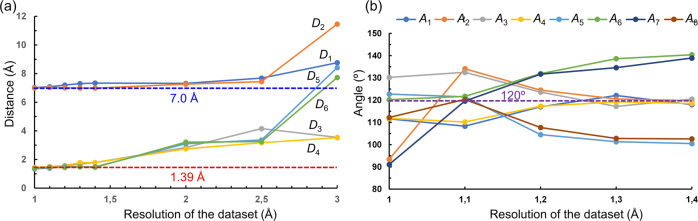
The trend of bond distances and angles in the structural models obtained using different data resolutions. **a** *D*_1_–*D*_6_ distances and **b** *A*_1_–*A*_8_ angles. Blue dash line: N···N distance in the model obtained by the dual-space method; red dash line: the ideal C…C bond distance in a phenyl ring; purple dash line: the ideal bond angle in a phenyl ring.

To further understand the reliability of structures solved by SA, we simulated datasets with different resolutions of 1.0, 1.1, 1.2, 1.3, 1.4, 2.0, 2.5, and 3.0 Å. During the data simulation, we excluded all reflections with *d*-values smaller than the specified resolutions, and scaled the intensity/sigma distribution similar to that in the experimental data (Supplementary Tables 1–9). With the resolution of 1.0–1.4 Å, we found that SA can provide reasonable structure solutions. The CF values are in the range of 0.590–0.609, which are similar to 0.596 as obtained from the experimental data of 0.9 Å resolution (Supplementary Table 10). On the aspect of framework geometry, the distortion becomes more severe with the decrease in data resolution. For example, the average difference in bond distances increased from 0.04 at 1.0 Å resolution to 0.22 Å at 1.4 Å resolution. On the other hand, the average difference in bond angles remains in the range of 7° to 12° (Fig. 4 and Supplementary Table 11). This shows that the decrease in resolution could affect the quality and reliability of structure solutions. When the resolutions are further reduced to 2.0, 2.5, and 3.0 Å, SA struggles to find a solution with reasonable geometry. The extent of tilting of the fragment was increased, as indicated by the increasing *D*_1_ and *D*_2_ distances with up to 8.76 and 11.46 Å from the data of 3.0 Å resolution. In addition, the poor identification of the positions of the C_4_ terminals resulted in large distances of *D*_3_–*D*_6_, which are in the range of 2.75–8.42 Å. As most of them increased beyond 3.0 Å, it is difficult to interpret the frameworks (Supplementary Fig. 2 and Supplementary Tables 11,  12).

## Discussion

We present a study by using SA to solve structures from COF nanocrystals, and compare the results obtained by SA to other structure solution methods. With high crystal quality of a resolution of 0.9 Å, the structural model of COF Py-1P solved by SA is in good agreement with the refined model obtained by the dual-space method. While the models provided by charge flipping and the classic direct method show similar atomic distribution, crystallographic details are lost. When the crystallinity of Py-1P is poor, as indicated by the 1.5 Å 3DED data resolution, it is possible to determine the unit cell parameters and space group. Using SA, we can obtain a structural model which contains distortions in the framework yet comparable to the reference model. This shows the possibilities to solve low-crystallinity COF structures by SA. In contrast, it is difficult to use the dual-space method, charge flipping, and classic direct method to solve the structure using low-resolution data. We further simulated data with different resolutions to study how they could affect the structure solutions given by SA. We found that when the resolution is higher than 1.5 Å, a good structural model can be obtained. However, when the resolution is reduced to lower than 2.0 Å, the distortion increases significantly, which prevents a meaningful interpretation of the framework structures. As the crystallization problem is a challenge not only for COFs, we anticipate that using SA for low-resolution 3DED data has potentials for determining structures of other framework materials such as metal-organic frameworks (MOFs), hydrogen-bonded frameworks (HOFs), zeolites, etc.

## Methods

### Material synthesis

We use the same Py-1P COFs as our previously reported, from which the detailed synthesis procedures can be found in ref. ^29^. In a typical synthesis, terephthalaldehyde (5.4 mg, 0.04 mmol) was dissolved in 1,4-dioxane (0.3 mL) and mixed with aniline (41 μL, 0.44 mmol) to form a clear solution. Aqueous acetic acid solution (90 μL, 6 M) was added to the mixture, leading to the formation of PPA. 10.0 mg (0.018 mmol) of DTA was dispersed in 0.5 mL of 1,4-dioxane, and the suspension was heated at 65 °C until a clear brown solution was obtained. Then, the heated solution of DTA was mixed with the solution containing PPA. The mixture was kept at 65 °C for a period of 1 month for growing Py-1P.

### 3DED data collection and data processing

The 3DED data were collected on a JEOL JEM2100 microscope and operated at 200 kV. A tomography holder with a tilting range of −70° to +70° was used. The selected-area aperture covers an area of c.a. 1.0 μm in diameter, and the electron diffraction patterns were recorded using a Timepix pixel detector QTPX-262k (512 × 512 pixels, pixel size 55 μm, Amsterdam Sci. Ins.). *Instamatic*^49^ was used for 3DED data collection, with every 20th frame being used to generate an image to trace the crystal. The goniometer rotation speed was set at 0.45° s^−1^, and the exposure time was set at 0.5 s per frame. Low-dose conditions were used to reduce the beam damage. The total tilting angle range is 92.2°. The 3DED data were processed with XDS package^50^. The dataset has a signal-to-noise ratio of 5.32 within the resolution of 1.50 Å. Due to the plate-like morphology of the crystal and the limitation of the goniometer tilting range, the data completeness is 0.425.

### Geometry optimization of monomers

The optimization of the geometries of the COF monomers was applied by using a pseudopotential plane-wave method within the DFT framework by the Accelrys Materials Studio package. After the creation of the initial structures of the monomers, their geometries were quickly optimized in the FORCITE module with the Dreiding forcefield of Materials Studio.

### Structure solution

The electron wavelength (0.02508 Å) and electron scattering factors were used. SA was conducted by using Sir 2019^47^. The number of runs were set as 10, with each run including 20 itineraries. Anti-bump restraints were used with the default values of 3.250 for C···N and 3.100 for N···N. Twenty parameters were refined during the SA process, which includes the translation and rotation of the rigid bodies, and the torsion angles within the rigid bodies. The dual-space method was conducted by using SHELXT^38^. The classic direct method was conducted by using SHELXS^48^. Charge flipping was conducted by using Superflip in JANA 2020^51^.

### 3DED data simulation

The Computational Crystallography Toolbox^52^ library was used to simulate data in similar conditions as they have reduced crystallinities. The experimental 3DED data with 0.9 Å resolution was used for the simulation. The resolution was first cut by excluding all reflections with *d*-values smaller than the desired resolution value. The data with resolution cutoffs are divided into the same number of resolution shells as in the original data. Then, we processed the hkl files and simulated sigma values in hkl files using the Computational Crystallography Toolbox library so that the distribution of I/sigma at each resolution shell matches with the distribution of that before the resolution cutoff.

## Supplementary information

Supplementary Information

## Data Availability

All data in this work are available within the article and the Supplementary Information. Additional reasonable requests for data supporting this publication should be addressed to the corresponding author.
