# Supplementary material for: Structure determination of a low-crystallinity covalent organic framework by three-dimensional electron diffraction
Source: Commun Chem. 2023 Jun 7;6:116. doi: 10.1038/s42004-023-00915-4 (PMC10247803; doi:10.1038/s42004-023-00915-4)
Supplement: Supplementary file 1 — Supplementary Information [file 42004_2023_915_MOESM1_ESM.pdf]

Supplementary Information for

Structure Determination of A Low-Crystallinity Covalent Organic Framework by Three-Dimensional Electron Diffraction

Guojun Zhou[1], Taimin Yang[1], and Zhehao Huang[1*]

[1]Department of Materials and Environmental Chemistry, Stockholm University, Stockholm SE-106 91, Sweden
*Email: zhehao.huang@mmk.su.se

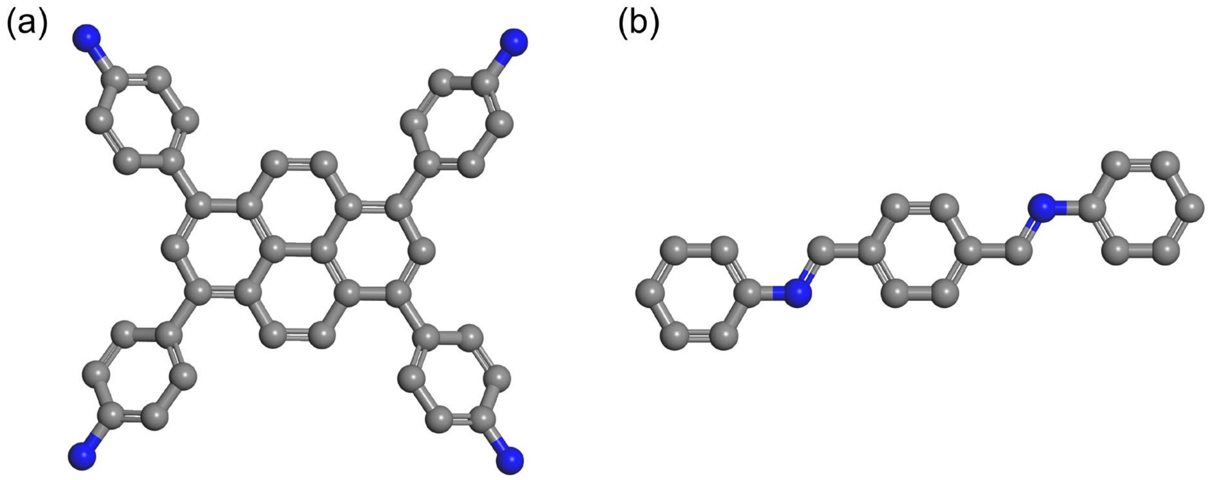

Supplementary Fig. 1: The structural model used to build the fragment for simulated annealing.

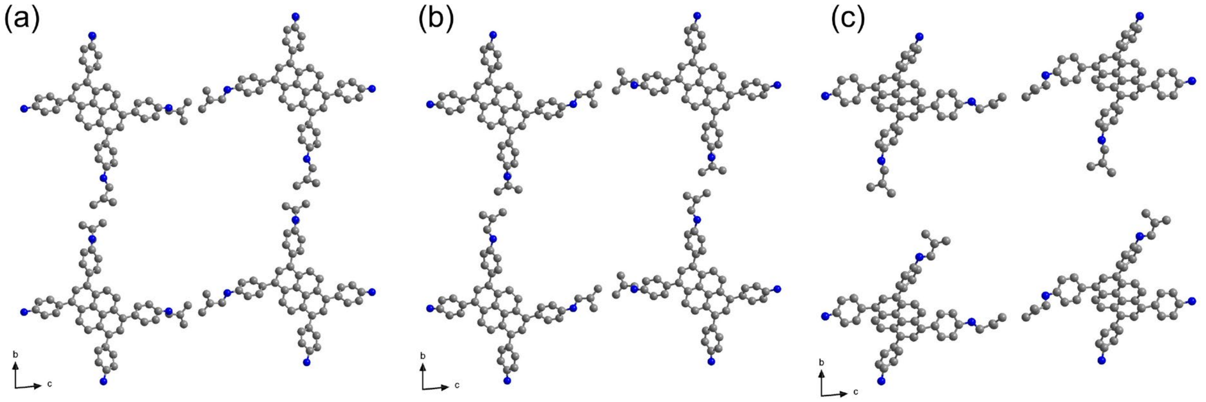

Supplementary Fig. 2: Structural models of Py-1P obtained using simulated 3DED data. Data resolutions of (a) 2.0 Å, (b) 2.5 Å, and (c) 3.0 Å. Significant distortion in the frameworks can be observed.

Supplementary Table 1: Experimental data statistics with the original resolution of 0.90 Å.

**Resolution (Å)**	**Mean I/sigma**
Inf - 2.60	8.65
2.60 - 2.00	8.26
2.00 - 1.72	6.42
1.72 - 1.56	5.77
1.56 - 1.45	4.49
1.45 - 1.37	4.55
1.37 - 1.30	4.08
1.30 - 1.24	4.12
1.24 - 1.19	4.14
1.19 - 1.15	3.47
1.15 - 1.11	3.34
1.11 - 1.07	3.66
1.07 - 1.04	3.65
1.04 - 1.01	2.80
1.01 - 0.98	1.74
0.98 - 0.95	1.09
0.95 - 0.93	0.78
0.93 - 0.90	0.40
**Overall (Inf - 0.90)**	3.85

Supplementary Table 2: Comparison of experimental data statistics with simulated data statistics after resolution cutoff to 1.00 Å.

**Resolution (Å)**	**Mean I/sigma**	
	**Experimental data**	**Simulated data**
Inf - 2.84	8.84	8.65
2.84 - 2.19	8.40	8.23
2.19 - 1.90	7.93	6.57
1.90 - 1.70	5.62	5.69
1.70 - 1.57	5.82	4.64
1.57 - 1.48	4.64	4.52
1.48 - 1.41	4.34	4.05
1.41 - 1.35	4.69	4.23
1.35 - 1.30	4.02	4.01
1.30 - 1.26	4.13	3.42
1.26 - 1.21	4.14	3.47
1.21 - 1.17	3.89	3.83
1.17 - 1.14	3.29	3.42
1.14 - 1.11	3.36	2.30
1.11 - 1.08	3.94	1.56
1.08 - 1.06	3.43	0.95
1.06 - 1.03	3.20	0.67
1.03 - 1.00	2.87	0.40
**Overall (Inf - 1.00)**	4.72	3.80

Supplementary Table 3: Comparison of experimental data statistics with simulated data statistics after resolution cutoff to 1.10 Å.

**Resolution (Å)**	**Mean I/sigma**	
	**Experimental data**	**Simulated data**
Inf - 3.14	9.13	8.66
3.14 - 2.45	8.15	8.20
2.45 - 2.09	8.59	6.42
2.09 - 1.87	7.71	5.81
1.87 - 1.73	5.33	4.44
1.73 - 1.61	6.17	4.61
1.61 - 1.53	4.82	4.08
1.53 - 1.47	4.79	4.28
1.47 - 1.41	4.12	4.00
1.41 - 1.36	4.84	3.51
1.36 - 1.32	4.31	3.50
1.32 - 1.28	3.87	3.60
1.28 - 1.24	4.09	3.38
1.24 - 1.20	4.26	2.53
1.20 - 1.17	3.67	1.52
1.17 - 1.15	3.31	0.93
1.15 - 1.12	3.35	0.67
1.12 - 1.10	3.31	0.40
**Overall (Inf - 1.10)**	5.15	3.88

Supplementary Table 4: Comparison of experimental data statistics with simulated data statistics after resolution cutoff to 1.20 Å.

Resolution (Å)	Mean I/sigma	
	Experimental data	Simulated data
Inf - 4.68	9.74	8.66
4.68 - 3.39	7.70	8.15
3.39 - 2.91	8.61	6.37
2.91 - 2.64	8.52	5.73
2.64 - 2.46	7.45	4.69
2.46 - 2.29	5.81	4.51
2.29 - 2.15	5.79	4.07
2.15 - 2.05	5.79	4.20
2.05 - 1.95	4.77	4.07
1.95 - 1.87	4.63	3.43
1.87 - 1.80	4.62	3.38
1.80 - 1.75	4.73	3.84
1.75 - 1.69	4.23	3.39
1.69 - 1.65	4.40	2.56
1.65 - 1.60	3.81	1.41
1.60 - 1.56	4.07	0.93
1.56 - 1.53	4.32	0.64
1.53 - 1.50	3.84	0.40
**Overall (Inf - 1.20)**	5.64	3.84

Supplementary Table 5: Comparison of experimental data statistics with simulated data statistics after resolution cutoff to 1.30 Å.

Resolution (Å)	Mean I/sigma	
	Experimental data	Simulated data
Inf - 3.47	9.74	8.69
3.47 - 2.66	8.11	8.20
2.66 - 2.31	8.00	6.32
2.31 - 2.06	8.68	5.70
2.06 - 1.90	8.67	4.52
1.90 - 1.77	7.68	4.54
1.77 - 1.67	7.03	4.13
1.67 - 1.59	4.98	4.03
1.59 - 1.53	5.89	4.01
1.53 - 1.48	5.85	3.52
1.48 - 1.43	5.26	3.58
1.43 - 1.39	4.29	3.56
1.39 - 1.35	4.81	3.54
1.35 - 1.32	4.36	2.33
1.32 - 1.29	4.73	1.50
1.29 - 1.26	4.30	0.96
1.26 - 1.23	4.44	0.66
1.23 - 1.20	3.68	0.40
**Overall (Inf - 1.20)**	6.05	3.80

Supplementary Table 6: Comparison of experimental data statistics with simulated data statistics after resolution cutoff to 1.40 Å.

Resolution (Å)	Mean I/sigma	
	Experimental data	Simulated data
Inf - 4.47	9.69	8.68
4.47 - 3.14	8.59	8.20
3.14 - 2.71	8.02	6.41
2.71 - 2.47	8.27	5.69
2.47 - 2.26	8.55	4.49
2.26 - 2.10	8.54	4.54
2.10 - 1.96	7.62	4.05
1.96 - 1.87	7.96	4.32
1.87 - 1.79	5.75	4.12
1.79 - 1.73	4.87	3.31
1.73 - 1.67	6.17	3.42
1.67 - 1.61	6.17	3.81
1.61 - 1.57	5.02	3.38
1.57 - 1.53	4.66	2.60
1.53 - 1.50	4.68	1.50
1.50 - 1.47	4.88	0.99
1.47 - 1.44	4.46	0.63
1.44 - 1.40	3.85	0.40
**Overall (Inf - 1.40)**	6.48	3.83

Supplementary Table 7: Comparison of experimental data statistics with simulated data statistics after resolution cutoff to 2.00 Å.

**Resolution (Å)**	**Mean I/sigma**	
	**Experimental data**	**Simulated data**
Inf - 6.22	8.35	8.64
6.22 - 4.89	10.35	8.12
4.89 - 3.95	10.37	6.38
3.95 - 3.59	10.21	5.77
3.59 - 3.19	7.90	4.57
3.19 - 2.97	6.36	4.54
2.97 - 2.85	8.66	4.10
2.85 - 2.72	8.21	4.13
2.72 - 2.60	7.68	3.90
2.60 - 2.49	8.27	3.40
2.49 - 2.40	9.91	3.50
2.40 - 2.31	8.25	3.67
2.31 - 2.24	8.13	3.50
2.24 - 2.17	7.41	2.45
2.17 - 2.11	9.32	1.58
2.11 - 2.06	9.23	1.06
2.06 - 2.00	6.69	0.54
**Overall (Inf - 2.00)**	8.50	3.86

Supplementary Table 8: Comparison of experimental data statistics with simulated data statistics after resolution cutoff to 2.50 Å.

**Resolution (Å)**	**Mean I/sigma**	
	**Experimental data**	**Simulated data**
Inf - 7.76	8.88	8.81
7.76 - 5.86	7.33	7.39
5.86 - 5.42	11.55	6.39
5.42 - 4.65	10.83	5.69
4.65 - 4.19	9.32	4.42
4.19 - 3.83	10.99	4.55
3.83 - 3.59	9.84	4.25
3.59 - 3.34	7.78	4.21
3.34 - 3.17	7.04	4.01
3.17 - 3.05	7.57	3.54
3.05 - 2.93	6.66	3.22
2.93 - 2.86	8.65	3.65
2.86 - 2.82	8.74	3.60
2.82 - 2.71	7.53	2.71
2.71 - 2.64	8.38	1.65
2.64 - 2.60	7.40	0.93
2.60 - 2.55	7.33	0.70
2.55 - 2.50	9.92	0.40
**Overall (Inf - 2.50)**	8.62	3.95

Supplementary Table 9: Comparison of experimental data statistics with simulated data statistics after resolution cutoff to 3.00 Å.

Resolution (Å)	Mean I/sigma	
	Experimental data	Simulated data
Inf -10.83	7.65	8.21
0.83 - 7.20	10.68	9.41
7.20 - 6.21	5.17	6.31
6.21 - 5.78	11.34	5.31
5.78 - 5.27	11.30	4.52
5.27 - 4.68	10.95	4.42
4.68 - 4.48	11.27	4.41
4.48 - 4.19	8.31	3.94
4.19 - 3.89	10.63	3.96
3.89 - 3.81	11.68	3.62
3.81 - 3.60	8.65	3.41
3.60 - 3.39	6.43	3.24
3.39 - 3.30	11.83	3.00
3.30 - 3.17	5.44	1.54
3.17 - 3.13	6.62	0.98
3.13 - 3.01	7.89	0.60
**Overall (Inf - 3.00)**	9.02	4.09

Supplementary Table 10: The cost function (CF) values of SA calculations using simulated 3DED datasets with different resolutions.

Data resolution	CF value
1.0 Å	0.598
1.1 Å	0.590
1.2 Å	0.598
1.3 Å	0.6091
1.4 Å	0.609
2.0 Å	0.612
2.5 Å	0.598
3.0 Å	0.554

Supplementary Table 11: Key distances and angles in the structural models obtained from SA using a data resolution ranges from 1.0 Å to 3.0 Å.

SA method	1.0 Å	1.1 Å	1.2 Å	1.3 Å	1.4 Å	2.0 Å	2.5 Å	3.0 Å
D_1 (Å)	7.02	7.08	7.20	7.30	7.34	7.32	7.69	8.76
D_2 (Å)	6.99	7.04	7.01	6.99	6.99	7.27	7.44	11.46
D_3 (Å)	1.42	1.51	1.58	1.67	1.79	2.84	4.15	3.54
D_4 (Å)	1.45	1.48	1.59	1.77	1.78	2.75	3.17	3.52
D_5 (Å)	1.35	1.39	1.51	1.50	1.49	3.10	3.36	8.42
D_6 (Å)	1.38	1.46	1.44	1.49	1.45	3.21	3.25	7.73
A_1 (°)	112	108	117	122	118	N/A	N/A	N/A
A_2 (°)	93	134	125	121	120	N/A	N/A	N/A
A_3 (°)	130	133	124	117	121	N/A	N/A	N/A
A_4 (°)	112	110	117	119	119	N/A	N/A	N/A
A_5 (°)	123	122	105	101	101	N/A	N/A	N/A
A_6 (°)	120	122	132	139	140	N/A	N/A	N/A
A_7 (°)	91	120	132	135	139	N/A	N/A	N/A
A_8 (°)	112	120	108	103	103	N/A	N/A	N/A

Supplementary Table 12: Comparison of key distances and angles in different structural models against those from the reference model. Differences of D_1 and D_2 are calculated against the refined reference model. Differences of D_3 - D_6 and A_1 - A_8 are calculated against optimal values in phenyl rings. All values are shown in absolute values.

Difference	1.0 Å	1.1 Å	1.2 Å	1.3 Å	1.4 Å	2.0 Å	2.5 Å	3.0 Å
D_1 (Å)	0.02	0.08	0.20	0.30	0.34	0.32	0.69	1.76
D_2 (Å)	0.04	0.01	0.02	0.04	0.04	0.24	0.41	4.43
D_3 (Å)	0.03	0.12	0.19	0.28	0.4	1.45	2.76	2.15
D_4 (Å)	0.06	0.09	0.2	0.38	0.39	1.36	1.78	2.13
D_5 (Å)	0.04	0	0.12	0.11	0.1	1.71	1.97	7.03
D_6 (Å)	0.01	0.07	0.05	0.1	0.06	1.82	1.86	6.34
A_1 (°)	8	12	3	2	2	N/A	N/A	N/A
A_2 (°)	27	14	5	1	0	N/A	N/A	N/A
A_3 (°)	10	13	4	3	1	N/A	N/A	N/A
A_4 (°)	8	10	3	1	1	N/A	N/A	N/A
A_5 (°)	3	0	15	19	19	N/A	N/A	N/A
A_6 (°)	0	2	12	19	20	N/A	N/A	N/A
A_7 (°)	29	0	12	15	19	N/A	N/A	N/A
A_8 (°)	8	0	12	17	17	N/A	N/A	N/A
